# Angiotensin receptor blocker Losartan inhibits tumor growth of colorectal cancer

**DOI:** 10.17179/excli2020-3083

**Published:** 2021-03-01

**Authors:** Milad Hashemzehi, Farzad Rahmani, Mahdieh Khoshakhlagh, Amir Avan, Fereshteh Asgharzadeh, Farnaz Barneh, Reyhaneh Moradi-Marjaneh, Atena Soleimani, Hamid Fiuji, Gordon A. Ferns, Mikhail Ryzhikov, Mohieddin Jafari, Majid Khazaei, Seyed Mahdi Hassanian

**Affiliations:** 1 Department of Medical Physiology, Faculty of Medicine, Mashhad University of Medical Sciences, Mashhad, Iran; 2 Iranshahr University of Medical Sciences, Iranshahr, Iran; 3 Tropical and Communicable Diseases Research Centre, Iranshahr University of Medical Sciences, Iranshahr, Iran; 4 Department of Clinical Biochemistry, Faculty of Medicine, Mashhad University of Medical Sciences, Mashhad, Iran; 5 Student Research Committee, Faculty of Medicine, Mashhad University of Medical Sciences, Mashhad, Iran; 6 Metabolic Syndrome Research Center, Mashhad University of Medical Sciences, Mashhad, Iran; 7 Medical Genetics Research Center, Mashhad University of Medical Sciences, Mashhad, Iran; 8 Faculty of Paramedical Sciences, Beheshti University of Medical Sciences, Tehran, Iran; Current address: Princess Maxima Center for Pediatric Oncology, 3584, CS, Utrecht, The Netherlands; 9 Brighton & Sussex Medical School, Division of Medical Education, Falmer, Brighton, Sussex BN1 9PH, UK; 10 Saint Louis University, School of Medicine, St. Louis, MO, USA; 11 Institute for Molecular Medicine Finland (FIMM), Helsinki Institute of Life Science, University of Helsinki, Finland

**Keywords:** colorectal cancer, Losartan, renin-angiotensin system

## Abstract

The renin-angiotensin system (RAS) is up-regulated in patients with colorectal cancer (CRC) and is reported to be associated with poor prognosis and chemo-resistance. Here we explored the therapeutic potential of targeting RAS in CRC using Losartan, an angiotensin receptor blocker. An integrative-systems biology approach was used to explore a proteome-level dataset of a gene signature that is modulated by Losartan. The anti-proliferative activity of Losartan was evaluated using 2- and 3-dimensional cell culture models. A xenograft model of colon cancer was used to investigate tumor growth with Losartan alone and in combination with 5-FU followed by histological staining (Hematoxylin & Eosin and Masson trichrome staining), biochemical analyses, gene expression analyses by RT-PCR, western blot/IHC, or MMP Gelatin Zymography studies. Effects on cell cycle and cell death were assessed by flow cytometry. Losartan inhibited cell growth and suppressed cell cycle progression, causing an increase in CRC cells in the G1 phase. Losartan significantly reduced tumor growth and enhanced tumor cell necrosis. An impact on the inflammatory response, including up-regulation of pro-inflammatory cytokines and chemokines in CRC cells are potential mechanisms that could partially explain Losartan's anti-proliferative effects. Moreover, metastasis and angiogenesis were reduced in Losartan-treated mice as observed by inhibited matrix metalloproteinase-2 and -9 activities and decreased tumor vasculature. These data demonstrate the therapeutic potential of combining chemotherapeutic regimens with Losartan to synergistically enhance its activity and target the renin-angiotensin system as a new approach in colorectal cancer treatment.

## Introduction

Colorectal cancer (CRC) is the third most common cancer and the fourth most common cause of cancer-related death globally (Marjaneh et al., 2018[37]). Inflammatory and hereditary diseases such as personal/family history of adenoma polyps and colorectal cancer, inflammatory bowel disease and ulcerative colitis, as well as life style, are associated with increased risk of colon cancer (Haggar and Boushey, 2009[24]). Progress in screening methods and introduction of novel specific inhibitors in the past decade incidence have decreased the mortality rate of CRC (Torre et al., 2015[50]). However, in metastatic CRC, the risk of recurrence remains a challenge in the clinical setting and finding an effective treatment is urgently needed. In the meantime, currently approved drugs may have undiscovered therapeutic properties and can be repurposed as adjunctive therapies to increase efficacy of current chemotherapies. For instance, several studies have shown that renin-angiotensin system inhibitors (RASIs) currently used as anti-hypertensive and renal protective medications may also exert anti-tumor activities (Neo et al., 2007[40]; Ahmadian et al., 2018[3]; Liang et al., 2008[35]). 

Whilst the renin-angiotensin system is mainly involved in the regulation of blood pressure and body fluid balance (Ager et al., 2008[1]), there are several studies showing that renin-angiotensin system also regulate different aspects of tumor progression, including: cell proliferation, angiogenesis, inflammation, and apoptosis (Deshayes and Nahmias, 2005[15]). Angiotensin converting enzyme (ACE) is one of the key enzymes in the renin-angiotensin system, converting angiotensin I to angiotensin II (Smith and Missailidis, 2004[47]). Angiotensin II is postulated to elicit tumorigenic effects by binding mainly angiotensin II receptor type I (ATR1) and angiotensin II receptor type II (ATRII) (Ager et al., 2008[1]). Interestingly, it has been shown that ACE inhibitors (ACEI) or Angiotensin II Receptor Blockers (ARBs) decrease tumor progression in several types of cancers. In support of these findings, it has been demonstrated that administration of ACEI or angiotensin II receptor blockers ARBs, reduce tumor burden, volume, and angiogenesis, and increase tumor apoptosis of CRC liver metastasis in a murine model (Neo et al., 2007[40]; Koh et al., 2014[30]). It has also been shown that exposure to ACEI and ARBs decrease tumor progression and hospitalization and increase survival in patients with advanced colon cancer (Engineer et al., 2013[20]). Thus, a greater understanding of the cytotoxic effects of Losartan on cancer cells may lead to novel and clinically beneficial applications of this potent angiotensin receptor antagonist in combination with chemotherapies in the clinic.

Here we have investigated the therapeutic potential and the molecular mechanism of Losartan in CRC progression by using a cellular system, animal model, and genome-wide alterations in the cellular pathways based on high throughput data deposited in the iLINCS (integrative Library of Integrated Network-Based Cellular Signatures) database for a better understanding and hence a better management of the disease.

## Materials and Methods

### Drugs and chemicals

Losartan and Fluorouracil (5-FU) were obtained from Sigma-Aldrich (Zwijndrecht, The Netherlands). F12/Dulbecco's Modified Eagle Medium (DMEM/F12), fetal bovine serum (FBS), penicillin and streptomycin were purchased from Gibco BRL, Life Technologies Inc. (Gaithersburg, MD, USA). Antibodies for phospho-AKT (Thr 308), PI3K (p110 α), and β-actin were obtained from Cell Signaling Technology (Beverly, MA, USA). Primary Cyclin D1 and secondary antibodies were purchased from Santa Cruz Biotechnology (Santa Cruz, CA, USA). 

### Systems analysis of Losartan effect on cellular pathways

The iLINCS database (http://www.ilincs.org/ilincs/) was used to extract the gene signature of 1-100 µM Losartan assessed on HT-29 colon cancer cell line datasets after 24 hour incubation with the drug (LINCSCP _126360, LINCSCP_126358, LINCSCP). Significantly up or down regulated genes were selected (Adjusted p-value < 0.05 and - 0.2 < log (expression) < 0.2). Genes were then submitted to the Enrichr database (http://amp.pharm.mssm.edu/Enrichr/) for pathway analysis (Kuleshov et al., 2016[32]). WikiPathways library was used as reference for pathways. An adjusted p-value of < 0.05 was chosen for identifying pathways for further analysis. The gene content of selected pathways was used in STRING v.10.5 for analysis of protein-protein interactions (Szklarczyk et al., 2015[49]). Interactions defined by text-mining were excluded and the resulting network was visualized with Gephi 0.8.2. Expression data related to the genes within selected pathways were presented as a heatmap using pheatmap package in R software.

### Cell culture

CT-26 cell line was obtained from the Pasteur Institute (Tehran, Iran) and maintained at 37 °C in 5 % CO_2_ in DMEM/F12 medium supplemented with 10 % heat-inactivated FBS and 0.1 % penicillin-streptomycin.

#### Cell viability (MTT) assay

MTT assay was used to determine cytotoxicity of different concentrations of Losartan (0-1000 μM) on CRC cells as previously described (Amerizadeh et al., 2018[5]).

### Multicellular spheroids

Spheroids were developed by seeding 10^5^ cells in RMPI/F12+GlutaMAX-I (1:1) into agarose coated 96-well plates. The spheroid formation, growth and Losartan cytotoxic effects were assessed over 7 days under an inverted phase contrast microscope Leica-DMI300B (Leica, Wetzlar, Germany). Spheroid volume (V) was calculated from the geometric mean of the perpendicular diameters D= (Dmax+Dmin)/2 as follows: V= (4/3) ×π (D/2)^3^.

### Cell cycle analysis

Flow cytometry was used for cell cycle analysis as described previously (Marjaneh et al., 2018[37]). Briefly, following treatment of CT26 cells with Losartan (300 and 500 nM) for 24 h, cells were exposed to propidium iodide (PI) and the cell cycle was analyzed using a FACSCalibur flow cytometer (BD Biosciences). 

### MMP gelatin zymography

The enzymatic activities of matrix metalloproteinases (MMP)-2 and -9 were studied using gelatin zymography as described previously (Bai et al., 2013[7]). Briefly, CT-26 cells were exposed to Losartan (300 nM) at different time points. The cell culture medium was collected and proteins at the same concentrations were electrophoresed on 10 % polyacrylamide zymogram gel containing 0.1 % gelatin. Following electrophoresis, the gels were subjected to a renaturation step overnight at 37 °C. Proteinase activities appeared as clear bands after Coomassie blue R-250 staining and de-staining. The intensity of bands was quantified using ImageJ software.

### Quantitative Real-Time Polymerase-Chain-Reaction (qRT-PCR)

mRNAs were extracted from CT-26 cells or CRC tissues and complementary DNAs (cDNA) synthesized as described (Dinarvand et al., 2015[16]; Hassanian et al., 2014[26]). The real-time PCR amplification was carried out using specific primers for target genes (Macrogene, Seoul, Korea) (Table 1(Tab. 1)) by light cycler Real-time PCR (Roche Diagnostics, Mannheim, Germany). Glyceraldehyde 3-phosphate dehydrogenase (GAPDH) was used as a housekeeping control gene. 

### Western blotting

Western blotting was performed as described (Hassanian et al., 2015[27], 2016[25]). Briefly, total protein of colon cancer tissues was extracted and equal amount of protein was electrophoresed on sodium dodecyl sulfate polyacrylamide gels (SDS-PAGE) and transferred to nitrocellulose membranes. Following blocking, membranes were incubated with different antibodies and bands were visualized using chemiluminescence substrates.

### Animal experiment

BALB/c mice were obtained from Pasteur Institute (Tehran, Iran) between 6-8 weeks old. Two weeks after implantation of tumor cells, when the tumor volume reached 80-100 mm^3^, mice were divided into 4 groups. Untreated mice were placed into group 1 as a control, group 2 received 5 mg/kg 5-FU every other day (i.p), group 3 was treated with 90 mg/kg Losartan every day (i.p), and group 4 received 5-FU/Losartan combination. Tumor size and volume were measured every other day. At the end of the experiment, day 14, tumor samples were harvested for histological staining and biochemical analysis.

### Histological assessment 

The harvested tumors were fixed, embedded in paraffin, and sectioned into appropriate thickness (5 μm). The de-paraffinized sections were stained with hematoxylin-eosin (H&E) or Masson trichrome. The necrotic area, tumor vessels and fibrosis area were evaluated.

### Immunohistochemistry assay

Immunohistochemistry staining for cluster of differentiation 31 (CD31), used primarily to identify endothelial cells, was performed as described (Mirzaei et al., 2018[38]). Briefly, mounted tissue sections were de-paraffinized and re-hydrated by using xylene and graded alcohols, respectively. Antigen retrieval was achieved by boiling slides in Tris/EDTA for 20 min. Tissue sections were incubated with diluted primary (1:50) and secondary (1:2000) antibodies. Next, the tissue sections were treated with DAB, counterstained with hematoxylin, and observed by light microscopy.

### Measurement of oxidative stress markers

The production of reactive oxygen species (ROS) was assessed using fluorescent 2′,7′-dichlorofluorescein diacetate (DCF-DA) according to manufacturer's instructions (Abcam, Cambridge, MA) (Negrei et al., 2016[39]). Catalase activity, nitric oxide (NO), malondialdehyde (MDA) and total thiol levels were measured in tissue homogenates as described previously (Amerizadeh et al., 2018[5]).

## Results

### Losartan affects various pathways related to progression of CRC progression

**Figure 1:**
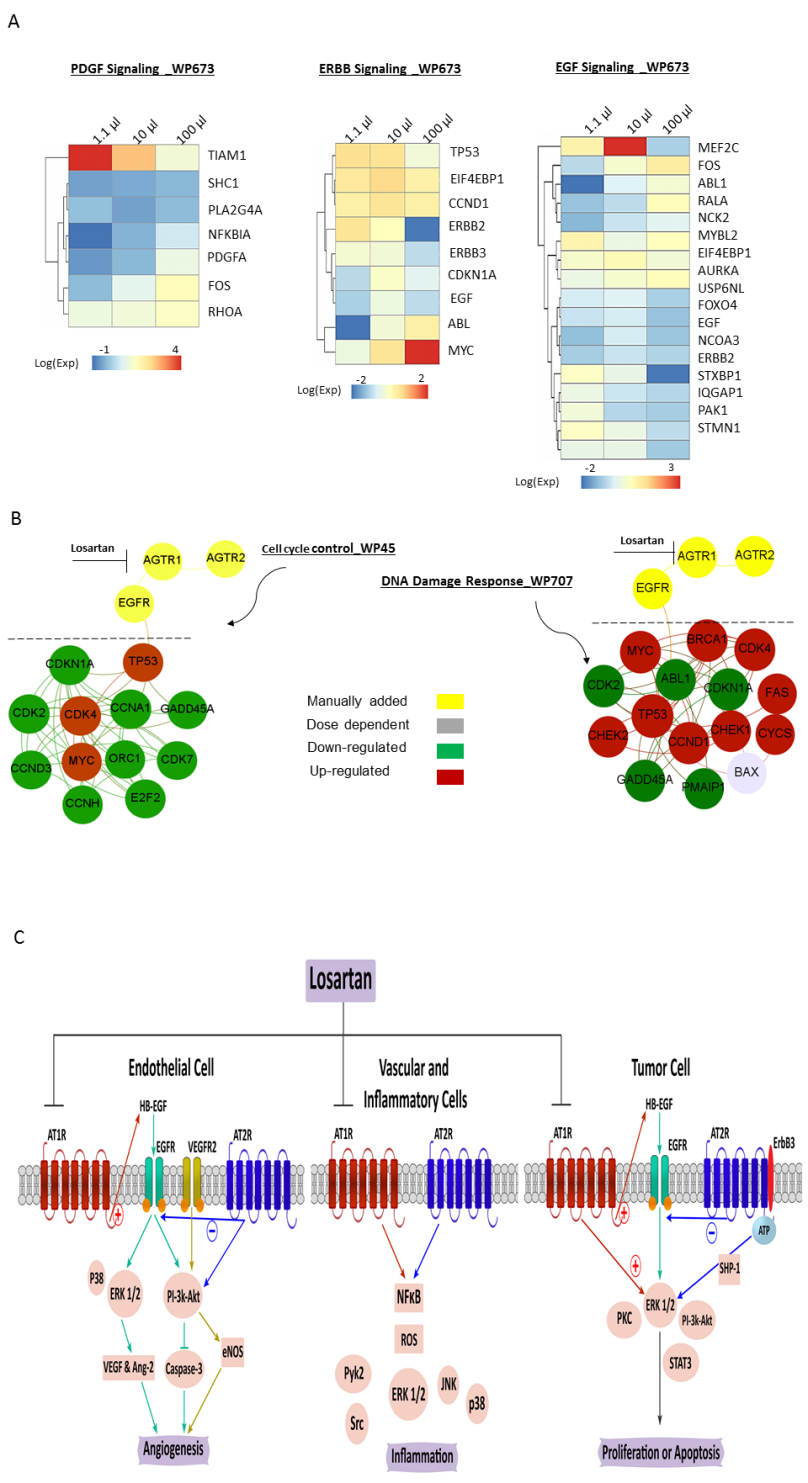
Analysis of significantly up- or down-regulated genes, enriched CRC-associated pathways, and network of protein-protein interactions following treatment of HT-29 colon cancer cell line extracted from iLINCS database. (A) Proliferative signaling pathway enrichment analysis carried out with Enrichr tool (library of WikiPathways) and subsequent gene expression changes within these pathways analyzed using iLINCS database in Losartan-stimulated HT-29 cells. The heat map shows the log (expression) level of genes in the selected pathways for different concentrations of losartan. (B) Gene contents of selected pathways associated with cell cycle were submitted to STRING v.10.5 for analysis of protein- protein interactions. Interactions defined by text-mining were excluded and the resulting network was visualized with Gephi 0.8.2. (C) Schematic representation of statistically enriched pathways involved in inflammatory, anti-angiogenic and anti-proliferative effects of Losartan against CRC

Following treatment of the HT-29 colon cancer cell line, analysis of significantly up- or down-regulated genes extracted from the iLINCS database showed that Losartan affects various pathways associated with tumor progression (using an adjusted p-value < 0.05). These intracellular pathways included ERBB, epidermal growth factor receptor (EGFR) and Platelet-derived growth factor receptor (PDGFR) pathways (Figure 1A(Fig. 1)), all of which regulate the PI3K-AKT signaling axis. Assessment of changes in gene expression also showed that Losartan affects the genes related to control of cell cycle progression and DNA damage response in HT29 colon cancer cell line. Several genes, including p53, BRCA1, and MYC were up-regulated while a number of Cyclin-dependent kinases were down-regulated (Figure 1A(Fig. 1)). The network of protein-protein interactions showed that these targets formed a highly interconnected network that is associated with ATR1 through EGFR (Figure 1B(Fig. 1)). Other statistically enriched pathways are categorized into angiogenesis and inflammation and are summarized in Figure 1C(Fig. 1), supporting Losartan's therapeutic properties in treating CRC progression by regulating cancer cell proliferation, apoptosis, angiogenesis and inflammation. 

Direct gene comparisons in the high throughput experiments may show cell dependent differences, while the altered cellular pathways are more robust indicators of drug effects on cells (Barneh et al., 2019[10]). We thus used the altered pathways obtained from the iLINCS data analysis as a starting point and further validated them in our experiments using CT-26 *in vitro* and* in vivo* models. 

### Losartan inhibits CT-26 cell viability

**Figure 2:**
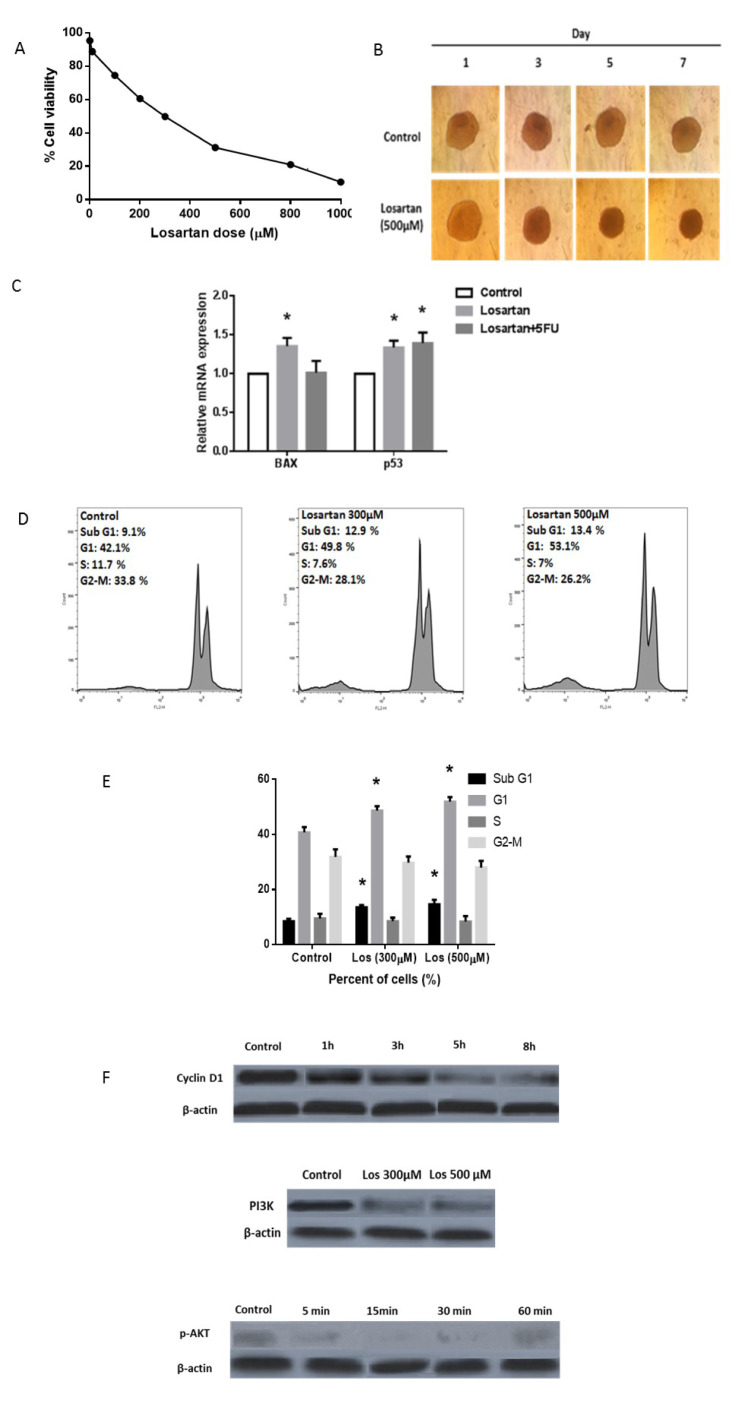
Losartan inhibits CT-26 cell proliferation and induces cellular apoptosis by regulating PI3K/AKT signaling pathway. (A) Inhibitory effects of Losartan (0-1000 μM) on CT-26 cell viability. (B) Cytotoxic effect of Losartan was investigated in a 3-D spheroid cell culture model system. (C) Losartan induces Bax and p53 mRNA expression in CRC tissues compared with control group. (D, E) Effects of Losartan treatment (for 24 h) on cell cycle progression in CT-26 cells. (F) Regulatory effects of Losartan on PI3K/AKT signaling pathway are determined by Western blotting. *P<0.05 comparison of Losartan and Losartan+5FU with control group

The MTT assay was used to determine cytotoxicity of different concentrations of Losartan (0-1000 μM) on CRC cells. As shown in Figure 2A(Fig. 2), Losartan decreased the CT-26 cell viability in a concentration-dependent manner with an IC_50_ of approximately 300 μM. To further assess the cytotoxic effects of Losartan on CRC cells, 3-D cell culture spheroids were treated with Losartan and tumor size and shape were analyzed for a week. Consistent with 2-D cell culture, Losartan significantly decreased spheroid size and induced tumor shrinkage in 3-D cell culture model (Figure 2B(Fig. 2)). Consistent with the *in silico* results, Losartan up-regulated mRNA levels of key pro-apoptotic genes including P53 and BAX in CT-26 cells (Figure 2C(Fig. 2)), suggesting that Losartan induces cell toxicity and apoptosis in CRC cells.

To further assess the cytotoxic effects of Losartan on CRC cells, CT-26 cells were exposed to different concentrations of Losartan (300, 500 μM) for 24 hours and cell cycle distribution was compared between groups. Losartan inhibited CRC cell progression by increasing percentage of G1 population from 37 % to 49 % (Figure 2D and E(Fig. 2)). It has been shown that cyclinD1 regulates the transition of cells from G1 to S phase (Resnitzky and Reed, 1995[45]; Baldin et al., 1993[8]). Moreover, cyclin D1 is regulated by phosphatidylinositol-4,5-bisphosphate 3-kinase (PI3K)/AKT signaling pathways (Ouyang et al., 2005[42]; Gao et al., 2004[22]; Canales et al., 2017[12]). To study the anti-proliferative mechanism of Losartan-mediated G1 arrest, we investigated the regulatory effect of Losartan on PI3K/AKT oncogenic signaling axis. As shown in Figure 2F(Fig. 2), in a time-dependent manner, Losartan significantly down-regulated expression of PI3K, AKT and their down-stream target, cyclin D1. These results clearly suggest that Losartan's anti-tumor activity is mediated by enhancing apoptosis and inhibition of cell proliferation in CRC cells.

### Losartan treatment inhibits tumor growth of colon cancer xenograft 

**Figure 3:**
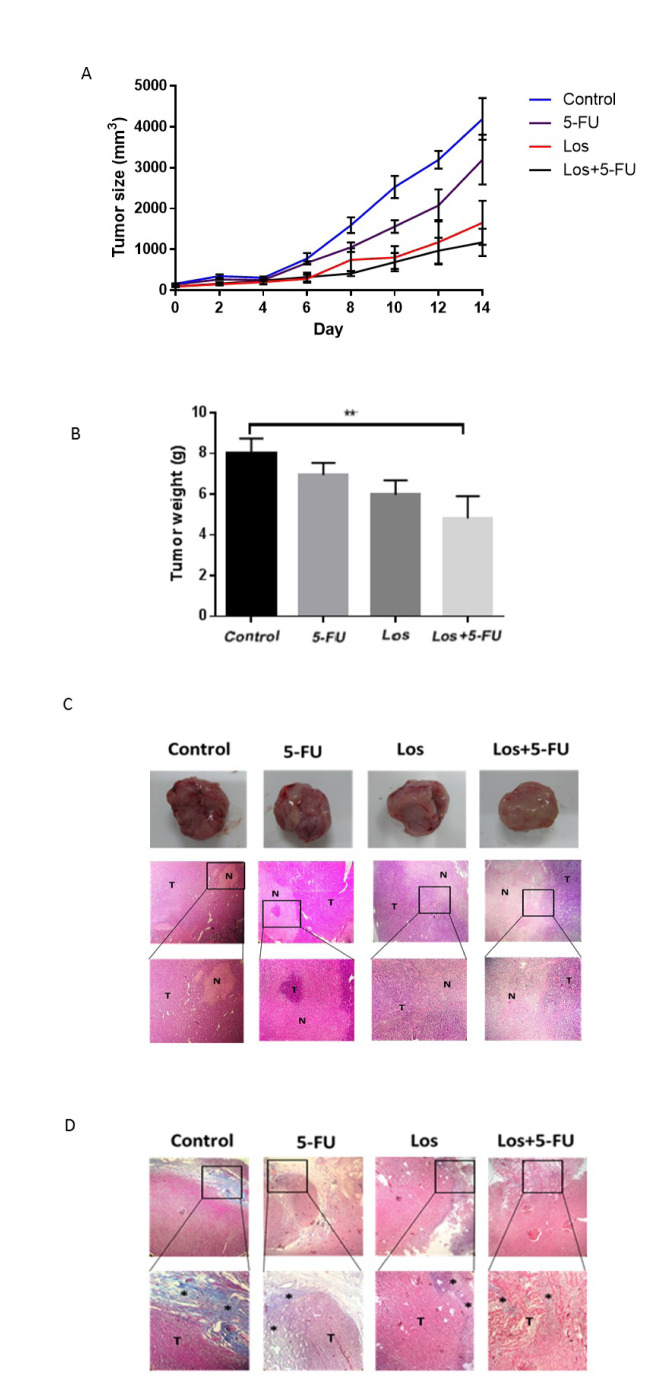
Losartan decreased tumor growth, size, weight, and fibrosis in CRC tumor xenografts. (A, B) Effect of Losartan on tumor size (A) and tumor weight (B) is compared beween groups. (C, D) Histological staining of tumor tissues by H&E and Masson trichrom for visualizing tissue necrosis (C) and tissue fibrosis (D), respectively. ** P<0.01 comparison of Losartan+5FU with control group

To validate our *in silico* and cellular findings, we investigated the effect of Losartan on tumor growth in CRC xenograft model. Consistent with above mentioned results, administration of Losartan significantly decreased tumor growth in murine CRC model and was well tolerated (Figure 3A(Fig. 3)). Interestingly, the suppressive effect of Losartan on tumor growth was more potent than 5-FU, the standard CRC chemotherapeutic, alone and combination therapy of losartan/5-FU, resulting in markedly greater decrease in tumor size (Figure 3A(Fig. 3)). Similarly, comparison of tumor weight between the groups showed that Losartan reduced tumor weight but this decrease was statistically significant only if co-administered with 5-FU (Figure 3B(Fig. 3)). Furthermore, histological staining of tumor tissues demonstrated that Losartan increased tissue necrosis (Figure 3C(Fig. 3)) and inhibited tissue fibrosis in the tumor xenografts (Figure 3D(Fig. 3)) as visualized by H&E and Masson trichrome staining, respectively. The murine model results suggest that effects of Losartan on tumor tissue necrosis and fibrosis are consistent with increased CT-26 cell toxicity, apoptosis and cell cycle inhibition in CRC.

### Losartan inhibits CRC progression by suppressing cell migration and angiogenesis

**Figure 4:**
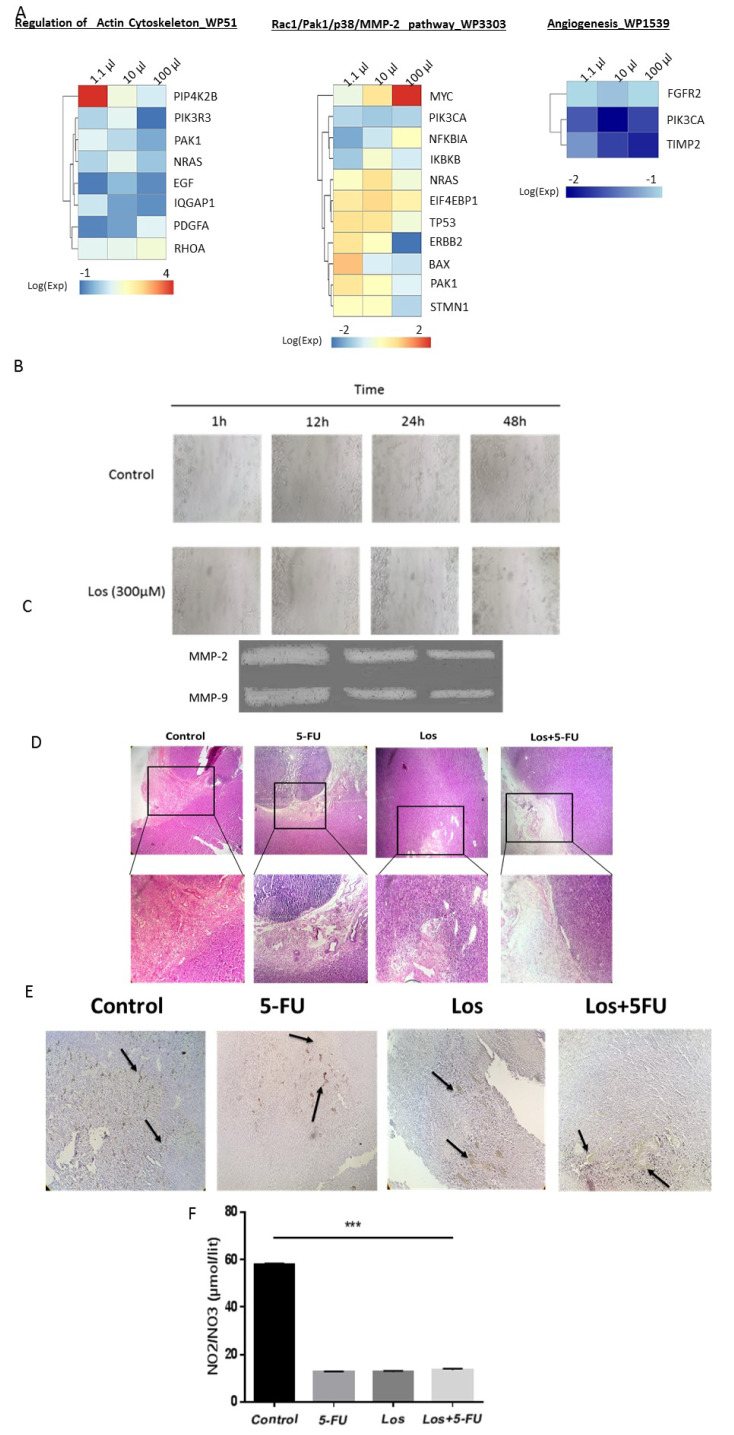
Losartan suppresses cellular migration and angiogenesis in CRC tissues samples. (A) For different concentrations of Losartan treatment in HT-29 colon cancer cell line, up- or down-regulated genes were extracted from the iLINCS database. Following pathway enrichment analysis, genes associated with migration were shown in the heatmaps. (B) CT-26 cells were treated with Losartan (300 μM) at different time-points and the anti-migratory effects of Losartan were measured. (C) The regulatory effects of Losartan on the activity of MMP 2,9 in CRC cells were investigated by zymography method. (D) The effects of Losartan on vascular density in CRC tissues were investigated using H&E staining. (E) CD31 positive cells and (F) nitrite levels are significantly decreased in losartan-treated mice, when compared to the control group. *** P<0.001 comparison of 5-FU, Losartan and Losartan+5FU with control group

Results extracted from the iLINCS database showed that angiogenesis is the pathway that is consistently down-regulated by increasing concentrations (1.11, 10 and 100 µM) of Losartan (adjusted p-value < 0.05) enriched by down-regulation of fibroblast growth factor receptor 2 (FGFR2), tissue inhibitor of metalloproteinases 2 (TIMP2) and phosphatidylinositol 4,5-bisphosphate 3-kinase catalytic alpha (PIK3CA) in HT-29 cells. Regulation of actin cytoskeleton and Rac-MMP pathway were also enriched with up/down regulated genes (Figure 4A(Fig. 4)), suggesting that Losartan can also regulate migration. Thus, the inhibitory effects of Losartan on migration and angiogenesis were evaluated. Results showed that Losartan (300 µM) decreased CT-26 cells migration (Figure 4B(Fig. 4)) and inhibited the enzymatic activities of MMP-2 and -9, key metastatic enzymes in CRC cells (Figure 4C(Fig. 4)).

Tumor vascularization plays a key role in the progression of a neoplasm from a localized tumor to a metastatic one (Folkman, 1971[21]; Liotta et al., 1974[36]). Our histological studies in tumor xenograft tissues showed that Losartan disrupts vascular development and morphogenesis, compared to control group (Figure 4D(Fig. 4)). Furthermore, expression levels of CD31 and NO, two pro-angiogenic factors, were studied in tumors and results indicated a significant decrease in both CD31 expression and NO levels in losartan-treated mice when compared to control (Figure 4E and F(Fig. 4)). These results suggest that Losartan elicits anti-tumor effects at least partially by inhibiting migration and angiogenesis in colon cancer cells.

### The effects of Losartan on oxidant/antioxidant balance and inflammation

**Figure 5:**
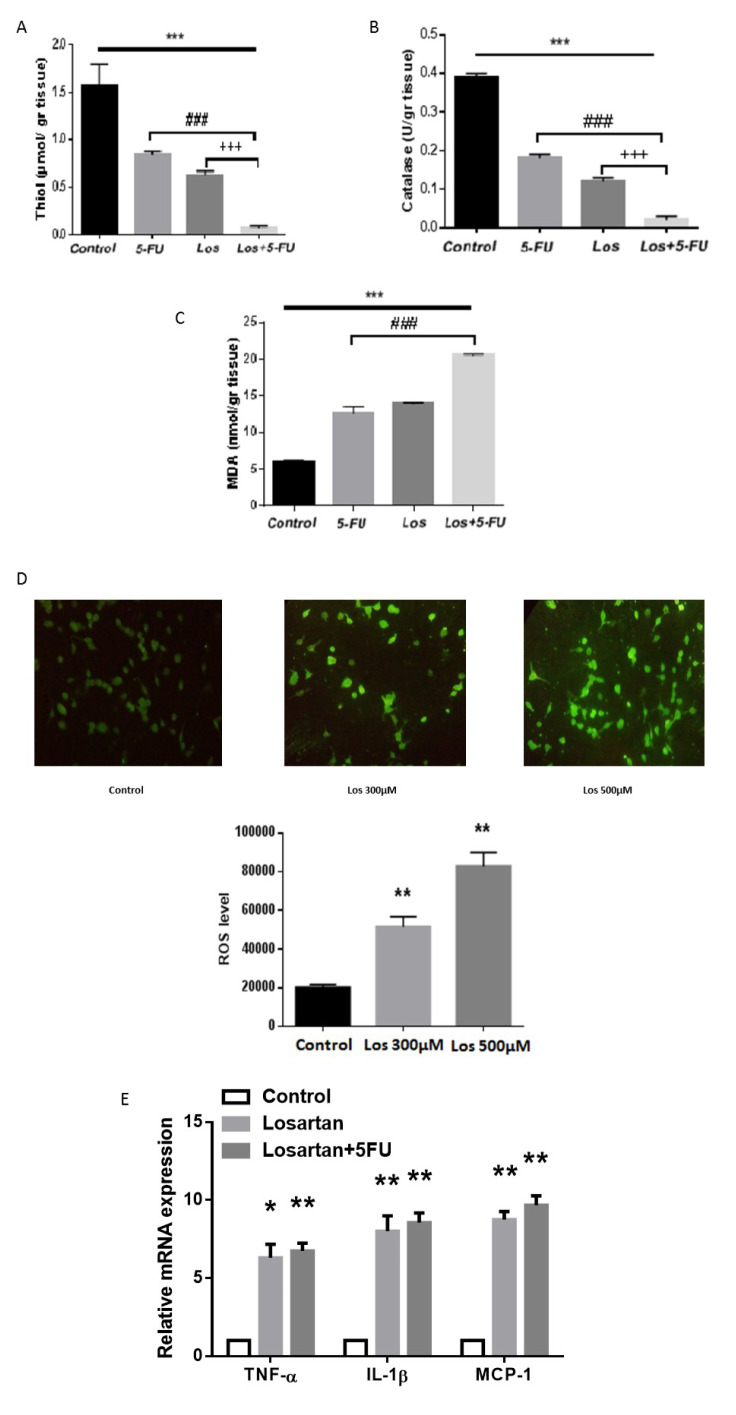
Losartan induces cellular oxidative stress and inflammatory responses in CRC tissues. (A) The regulatory effect of Losartan on total thiol concentrations was measured in tissue homogenates. (B) The same as A except that catalase activity was measured. (C) The same as A except that MDA level was compared between groups. (D) Losartan significantly induces cellular oxidative stress in CT-26 cells. (E) mRNA levels of pro-inflammatory cytokines were investigated by q-PCR in CRC tissues. * P<0.05, ** P<0.01 and *** P<0.001 comparison of 5-FU, Losartan and Losartan+5FU with control group, ### P<0.001 comparison of Losartan+5FU with 5-FU group, +++ P<0.001 comparison of Losartan+ 5FU with Losartan group

Next, we investigated the effects of Losartan on inflammation by measuring oxidant/antioxidant balance in tissue homogenates. We showed that in tumor tissues Losartan decreased thiol concentrations and catalase activity, two anti-oxidant markers (Figure 5A and B(Fig. 5)), and increased MDA level and ROS generation, biomarkers of oxidative stress (Figure 5C and D(Fig. 5)). Further studies showed that the effect of 5-FU on oxidant/ anti-oxidant balance was the same as Losartan and co-administration of losartan/5-FU elicited an additive effect in regulating oxidative stress in tumor tissue homogenates. 

To further identify the regulatory effects of Losartan on cellular pro-inflammatory responses, we evaluated the expression of pro-inflammatory molecules in losartan-stimulated CRC cells. Our results showed that Losartan treatment significantly increased the expression of IL-1β, TNF-α, and MCP-1 compared to the control group (Figure 5E(Fig. 5)). These data suggest that the anti-tumor activity of Losartan against colon cancer cells may be mediated by disruption of the oxidant/antioxidant balance as well as up-regulation of pro-inflammatory cytokines, leading to increased cancer cell death.

See also the Supplementary data.

## Discussion

The renin-angiotensin system plays an important role in cancer progression in different malignancies. In this study, we integrated systems and molecular biology to investigate the mechanisms of the anti-tumor effects of Losartan in CRC progression in cellular and animal models. Our results showed that Losartan elicits potent anti-tumor properties by inhibiting cell proliferation, migration, inflammation, and angiogenesis while increasing apoptosis in CRC.

Aberrant activation of renin-angiotensin system is found in different malignancies and is associated with tumor metastasis, invasion, migration, and angiogenesis (Ager et al., 2008[1]; Suganuma et al., 2005[48]; Juillerat-Jeanneret et al., 2004[28]; Kikkawa et al., 2004[29]; Dinh et al., 2001[17]). The activated form of AT1R triggers the Ras/PI3K/AKT/mTOR axis, a key proliferative signaling pathway in tumors (Baldus et al., 2010[9]; Du et al., 2012[18]). Li et al. have also shown that the inhibition of AT1R significantly decreased AKT activation whereas Angiotensin II up-regulated mTOR in esophageal carcinoma cells (Li et al., 2016[34]). Cyclin D1, a key downstream target of PI3K/AKT/ mTOR signaling, is over-expressed in several human cancers including CRC (Rahmani et al., 2018[44]; Arqués et al., 2016[6]). Cyclin D1 is a regulatory subunit for cyclin-dependent kinases including CDK4 and plays a prominent role in cell cycle progression from G1 to S phase (Peurala et al., 2013[43]). In the current study, we explored whether proliferative effects of angiotensin on CRC cells can be suppressed by Losartan by investigating downstream targets of Angiotensin/AT1R signaling, such as PI3K/AKT/ mTOR pathway activity and cyclin D1 expression. Our findings indicate that Losartan treatment inhibits PI3K/AKT signaling as visualized by down-regulation of PI3K and Cyclin D1 protein levels and inhibition of AKT phosphorylation.

Further studies showed that activation of PI3K/AKT signaling correlates with cancer cell apoptosis (Brader and Eccles, 2004[11]; Osaki et al., 2004[41]). AKT inhibited apoptosis through the phosphorylation of pro-apoptotic proteins (Cardone et al., 1998[13]; Datta et al., 1997[14]). Zhao et al. showed that Losartan induced apoptosis through the inhibition of PI3K/ AKT pathway in MCF-7 cell line (Zhao et al., 2008[52]). P53 and BAX are key pro-apoptotic regulators in apoptosis process (Ruvolo et al., 2001[46]; Li et al., 2009[33]). Furthermore, Ahmadian el al. demonstrated that Azilsartan, an AT1R antagonist, elevated apoptosis at least partially by increasing BAX mRNA expression in HepG2 hepatocellular carcinoma cells (Ahmadian et al., 2018[3]). Moreover, Koh et al. showed that RAS inhibitors induced apoptosis in colorectal cancer liver metastases (Koh et al., 2014[30]). In agreement with these results, we demonstrated that Losartan induces apoptosis by inhibition of the PI3K/AKT pathway and elevation of p53 and BAX levels in CRC. 

We also evaluated the anti-tumor effects of Losartan in a mouse model of colon cancer. Our results indicated that Losartan decreased tumor growth by inhibiting angiogenesis and changing the oxidant/anti-oxidant balance in tumor tissue. There is evidence that renin-angiotensin system inhibitors suppress tumor angiogenesis by reducing vascular endothelial growth factor expression and altering the tumor microenvironment (Greene and Amaral, 2002[23]; Kubota et al., 2011[31]). Similarly, Valuckaite et al. showed that Losartan decreased angiogenesis by reducing VEGF protein levels and VEGF expression in AOM induced colorectal cancer (Valuckaite et al., 2015[51]). Neo et al. have shown that renin-angiotensin system blockers, captopril and irbesartan, significantly decreased tumor growth in colorectal cancer liver metastases which was correlated with reduced central microvascular density (Neo et al., 2007[40]). In line with these findings Koh et al. demonstrated that captopril, a renin-angiotensin blocker, decreased angiogenesis by reducing CD-34-positive vessels in CRC liver metastases (Koh et al., 2014[30]). Our histological studies in tumor xenograft tissues showed that Losartan disrupts vascular development and morphogenesis, and down-regulates levels of CD31 and NO, two pro-angiogenic factors, when compared to the control group. 

There is some evidence that the current drugs used for cancer treatment affect oxidative stress. ROS production has an important role in apoptosis induction and reduction of cell viability in early stages of cancer (Negrei et al., 2016[39]; Eftekhari et al., 2018[19]). Furthermore, elevation of ROS leads to changing of mitochondrial permeability transition pore and elevation of cytochrome C-induced apoptosis (Ahmadian et al., 2018[4], 2016[2]). Ahmadian et al. demonstrated that azilsartan induced ROS formation in HepG2 hepatocellular carcinoma cells (Ahmadian et al., 2018[3]). Results of our study showed that administration of Losartan increased MDA levels and reduced total thiol concentration and catalase activity. These results suggest that alterations of the oxidant-antioxidant status can be one of the underlying mechanisms in Losartan anti-tumor activities against colon cancer cells. 

Taken together, we have shown that Losartan elicits its anti-tumor properties by inhibiting CRC cell growth, cell cycle progression, reducing angiogenesis and attenuating migration. In addition, it enhances tumor cell necrosis, elevating inflammatory responses, altering oxidant/anti-oxidant balance, and inducing apoptosis. Combination of Losartan and 5-FU revealed synergistic and additive anti-tumorigenic properties, suggesting that targeting of the renin-angiotensin system presents a potentially new therapeutic strategy in colon cancer treatment.

## Notes

Milad Hashemzehi, Farzad Rahmani, Mahdieh Khoshakhlagh and Amir Avan contributed equally as first authors.

Majid Khazaei and Seyed Mahdi Hassanian (Department of Clinical Biochemistry, Faculty of Medicine, Mashhad University of Medical Sciences, Mashhad, Iran; Phone: (+98) 5138002227, Fax: (+98) 5138002389, E-mail: HasanianmehrM@mums.ac.ir) contributed equally as corresponding authors.

## Disclosure

The authors declare that they have no conflict of interest.

## Funding

This study was supported by grants awarded by the Mashhad University of Medical Sciences (Grant No. 961748) and the Biotechnology Development Council of the Islamic Republic of Iran (Grant No: 961110). 

## Supplementary Material

Supplementary data

## Figures and Tables

**Table 1:**
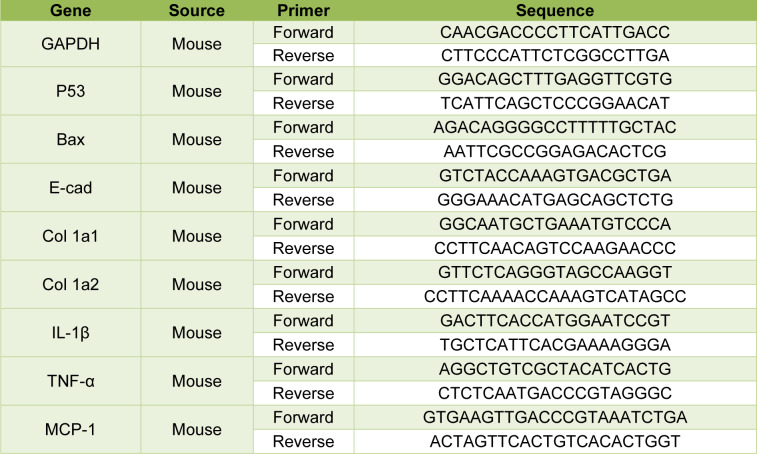
qPCR primer sequences
